# Proteomic Screening for Prediction and Design of Antimicrobial Peptides with AmpGram

**DOI:** 10.3390/ijms21124310

**Published:** 2020-06-17

**Authors:** Michał Burdukiewicz, Katarzyna Sidorczuk, Dominik Rafacz, Filip Pietluch, Jarosław Chilimoniuk, Stefan Rödiger, Przemysław Gagat

**Affiliations:** 1 Faculty of Mathematics and Information Science, Warsaw University of Technology, 00-662 Warsaw, Poland; michalburdukiewicz@gmail.com (M.B.); dominikrafacz@gmail.com (D.R.); 2 Department of Bioinformatics and Genomics, Faculty of Biotechnology, University of Wrocław, 50-383 Wrocław, Poland; sidorczuk.katarzyna17@gmail.com (K.S.); fpietluch@gmail.com (F.P.); jaroslaw.chilimoniuk@gmail.com (J.C.); 3 Faculty of Natural Sciences, Brandenburg University of Technology Cottbus-Senftenberg, 01968 Senftenberg, Germany; stefan.roediger@b-tu.de; 4 Faculty of Health Sciences, Joint Faculty of the Brandenburg University of Technology Cottbus-Senftenberg, the Brandenburg Medical School Theodor Fontane and the University of Potsdam, 01968 Senftenberg, Germany

**Keywords:** AMP, antimicrobial peptides, host defense peptides, multidrug-resistant bacteria, prediction, proteomic screening, random forest

## Abstract

Antimicrobial peptides (AMPs) are molecules widespread in all branches of the tree of life that participate in host defense and/or microbial competition. Due to their positive charge, hydrophobicity and amphipathicity, they preferentially disrupt negatively charged bacterial membranes. AMPs are considered an important alternative to traditional antibiotics, especially at the time when multidrug-resistant bacteria being on the rise. Therefore, to reduce the costs of experimental research, robust computational tools for AMP prediction and identification of the best AMP candidates are essential. AmpGram is our novel tool for AMP prediction; it outperforms top-ranking AMP classifiers, including AMPScanner, CAMPR3R and iAMPpred. It is the first AMP prediction tool created for longer AMPs and for high-throughput proteomic screening. AmpGram prediction reliability was confirmed on the example of lactoferrin and thrombin. The former is a well known antimicrobial protein and the latter a cryptic one. Both proteins produce (after protease treatment) functional AMPs that have been experimentally validated at molecular level. The lactoferrin and thrombin AMPs were located in the antimicrobial regions clearly detected by AmpGram. Moreover, AmpGram also provides a list of shot 10 amino acid fragments in the antimicrobial regions, along with their probability predictions; these can be used for further studies and the rational design of new AMPs. AmpGram is available as a web-server, and an easy-to-use R package for proteomic analysis at CRAN repository.

## 1. Introduction

Abuse and overuse of antibiotics in human health care and animal breeding has greatly contributed to a worldwide resistance to antibiotics. Moreover, the fact that hardly any new classes of antibiotics have been introduced to the market for decades makes the situation even more alarming [1,2]. Multidrug-resistant bacteria, the so-called ‘superbugs’, threaten our ability to tackle even common infectious diseases, resulting in prolonged illnesses and death of tens of thousands of people only in Europe and the United States [3,4]. Therefore, a lot of effort is being made to develop new antimicrobial agents, including antimicrobial peptides (AMPs).

AMPs, also known as cationic host defense peptides, are molecules that are widespread in all branches of the tree of life [5]. In multicellular organisms, they participate in the first line of defense against bacteria, fungi, protozoans and viruses, and can even target cancer cells [6,7]. In addition to their microbicidal, antiviral and antitumor activities, they have immunomodulatory properties and may have a role in autoimmune disorders, wound healing and angiogenesis [8,9,10]. In microorganisms, i.e., unicellular eukaryotes, bacteria and archaeans, they are used for self-protection and microbial competition [11,12,13].

AMPs are short, generally fewer than 50 amino acids, rich in positive and hydrophobic residues, which gives them an amphipathic character, and do not display any similarities in sequence composition [14,15,16]. Due to their structural characteristics, AMPs preferentially interact with negatively charged components of bacterial membranes, but do not adversely affect other eukaryoticmembranes. The latter are rich in neutral phospholipids and stabilizing cholesterol. AMPs act on the lipid bilayer in a detergent-like manner, solubilizing it into micelles, and/or penetrating it by forming pores. Both interactions lead to transient membrane permeabilization and cytoplasmic leakage that, depending on the AMP concentration, may trigger cell death [14,16,17,18]. Another AMP mechanism for efficient microbial killing is to act intracellularly, thus inhibiting, e.g., proteases, cell division and biosynthesis of proteins, nucleic acids and components of the cell wall [19]. The interaction of AMPs with so many components of the bacterial cell gives them an advantage over traditional antibiotics, i.e., makes them less prone to select for resistance [20,21,22]. Taking into account the therapeutic potential of AMPs [16,23] and the fact that superbugs are on the rise [1,2], it is of vital importance to find new AMPs. Unfortunately, the experimental procedures to identify novel AMPs are time-consuming, expensive and, most importantly, ineffective given the staggering numbers of genomes being sequenced. Consequently, there is a demand for high throughput computational tools to screen proteomes for AMPs with high accuracy.

A variety of computational approaches have been adopted for AMP prediction, and over the years, the performance of new computational tools have gradually improved with machine learning algorithms in the lead. Many of the AMP prediction methods have also been made accessible as web servers and the top-ranking ones already accept multiple query sequences, e.g., AMPScanner [24], ADAM [25], iAMP-2L [26], CAMPR3 [27] and iAMPpred [28]. However, there are still challenges to overcome, such as the prediction of longer AMPs. In their comparison of AMP prediction tools, Gabere and Noble [29] indicated that many investigated classifiers, including accessible at that time ADAM [25] and CAMPR3 [27], exhibited strong length dependence, i.e., assigned very high scores for sequences over 100 amino acids regardless of whether they were AMPs or not.

In practice, there are plenty of longer peptides that do exhibit antimicrobial properties (Table 1), e.g., milk proteins, such as α-lactoglobulin, β-lactoglobulin or lactoferrin [30,31,32,33]. The latter is especially of great importance, as literally hundreds of papers have been dedicated to its antimicrobial and antitumor activities. It is an 80 kDa iron-binding glycoprotein that, upon proteolitic processing, produces functional AMPs, such as 11 amino acid N-terminal lactoferrin fragment, lactoferricin (lactoferrin fragment 17–41) and lactoferrampin (lactoferrin fragment 268–284) [32]. An important source of antimicrobial peptides and proteins are also animal venomes [34,35]. An example of such a protein is L-amino oxidase from pit vipers that upon proteolytic cleavage, similarily to lactoferrin, generates three functional AMPs [36]. The most interesting are, however, proteins that do not exhibit any antimicrobial activities, such as human thrombin, but the products of their proteolysis do; they can be called ‘cryptic’ AMPs. In the case of human thrombin, its C-terminal peptides (527–622, 597–622, 604–622) constitute a novel class of AMPs produced during wounding and with therapeutic potential against infection and septic shock [37].

Our goal was to launch a high-throughput computational classifier, AmpGram, that could efficiently scan proteomes not only for typical AMPs but also longer proteins with AMP properties, including cryptic AMPs, and to indicate with high accuracy regions responsible for the AMP activity. AmpGram uses n-grams (amino-acid motifs) and random forests (a machine learning method) as an AMP classification algorithm. This methodology has already been used with success in our previous projects to create software for prediction of amyloid proteins [40], signal peptides, [41] and to assess optimal growth conditions for methanogens [42].

A new approach that identifies protein AMP potential regions is needed, not only because of the alarming situation with the growing bacterial resistance but, because small peptides are easier and cheaper to synthesize and present fewer side effects as indicated, e.g., by pardaxin [43]. Moreover, their activity can be easily improved by sequence modification that increases hydrophobicity and/or positive charge. Application of n-grams also allowed us to overcome the problem of high score–length dependency [29]. The overprediction for longer AMPs could not have been solved by simply their inclusion in the positive training dataset because their amino acid composition is hardly distinguishable from other proteins in contrast to typical AMPs (Supplementary Figure S1). The similarity in amino acid composition between longer AMPs and the negative dataset results from the fact that only short regions of proteins are responsible for their AMP properties.

## 2. Results and Discussion

### 2.1. Benchmark Analysis of AMP Predictors

**Figure 1:**
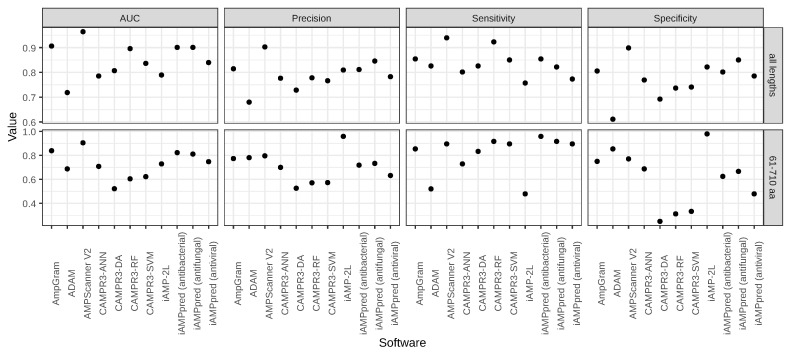
Comparison of AmpGram performance with other top-ranking predictors.

The benchmark analysis involved AmpGram and other top-ranking AMP predictors: AMPScanner [24], ADAM [25], iAMP-2L [26], CAMPR3 [27] and iAMPpred [28]. In order to compare their performance, the values of AUC (the Area Under the ROC receiver operating characteristic—Curve), precision, sensitivity and specificity were calculated for the test dataset. The performance results include the division of the benchmark dataset into five groups according to the sequence length (for details, see Section 3). However, to keep the article concise only the results for (i) all lengths and (ii) the longest AMPs are presented. The group of all lengths is dominated by shorter sequences, from ten to 60 amino acids, i.e., typical AMPs, and therefore biased against longer peptides and proteins. Consequently, the results in Figure 1 and Table 2 and Table 3 include the most informative groups analyzed. The complete results of the research are available in the Supplementary Materials (Figure S2 and Tables S1–S4).

**Figure 2:**
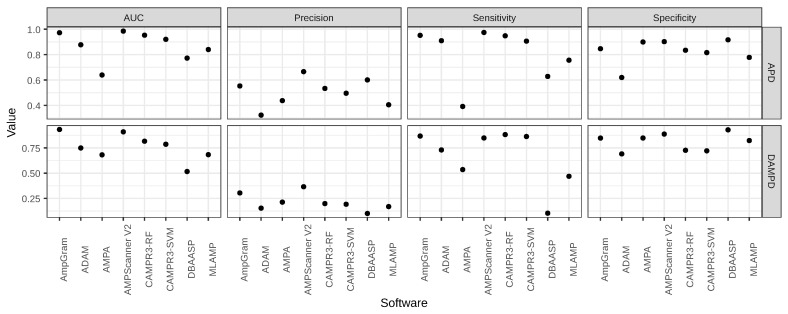
Comparison of AmpGram and AMPscanner [24] performance on the APD and DAMPD dataset with other predictors from Gabere and Noble’s benchmark and according to their methodology [29]. Sequences used to train either AmpGram or AMPScanner were removed from the DAMPD dataset. The benchmark without their removal is presented in Figure S3 in the Supplementary Materials. The very low values of precision are due to the very large negative dataset used (for details, see Section 3).

The benchmark results (Figure 1, Table 2 and Table 3) confirm that AmpGram performs very well but it is outperformed by AMPScanner [24], both for the group of all lengths (AUC: 0.964 vs. 0.906) and the longest AMPs (AUC: 0.905 vs. 0.839). However, the benchmark is biased against AmpGram because our test dataset could contain sequences that were included in the training datasets of other AMP predictors, including AMPScanner [24]. In order to test the influence of the benchmark bias, we compared the performance of AmpGram and AMPScanner on two datasets: APD3 [44] and DAMPD [45] in accordance with the methodology by Gabere and Noble [29]. It is important to emphasize that AMPScanner [24] was exclusively trained on sequences from the APD3 database [44], and neither AMPScanner [24] nor AmpGram used the DAMPD database [45]. To ensure that the DAMPD dataset [29] is indeed unbiased, we have additionally searched it and removed all sequences that were present in the AmpGram or AMPScanner [24] training dataset. As expected, AMPScanner beats AmpGram on the biased APD3 dataset (AUC: 0.985 vs. 0.972; Figure 2, Table 4); however, AmpGram outperforms AMPScanner [24] on the unbiased DAMPD dataset (AUC: 0.932 vs. 0.909; Figure 2, Table 5). This indicates that AmpGram is a more robust predictor. Moreover, in contrast to AMPScanner [24], AmpGram also allows query sequences to contain non-standard amino acids.

The other top-ranking AMP classifiers are not far behind AmpGram in the prediction of typical AMPs, but they have problems with longer peptides and proteins (Figure 1, Table 2 and Table 3), e.g., all CAMPR3 tools [27], which are based on: random forests (CAMPR3-RF), support vector machine (CAMPR3-SVM), artificial neural network (CAMPR3-ANN) and discriminant analysis (CAMPR3-DA), are characterized by decent sensitivity but very low specificity and precision. Sensitivity and specificity reflect the proportion of AMP and non-AMP sequences that are identified correctly as AMPs and non-AMPs, respectively, and precision the proportion of AMPs that actually are AMPs [46,47]. It means that all CAMPR3 algorithms, tend to ‘overpredict’ longer sequences as AMPs, i.e., generate a high number of false positive results. This high score–length dependency has already been indicated by Gabere and Noble [29] and also concerns iAMPpred [28]. In contrast to CAMPR3 and iAMPpred, ADAM [25] has very low sensitivity, and decent specificity and precision, which means that the program rather ‘underpredictis’ longer peptides and proteins, i.e., generates a high number of false negative results.

### 2.2. Prediction of Potential AMP Regions and Fragments

The goal behind development of AmpGram was to introduce a high throughput and accurate computational classifier that could search proteomes not only for typical AMPs, but also longer and cryptic AMPs, such as lactoferrin [32] and thrombin [37], respectively. Cryptic AMPs represent AMP sequences embedded in proteins that do not seem to have any AMP properties.

**Figure 3:**
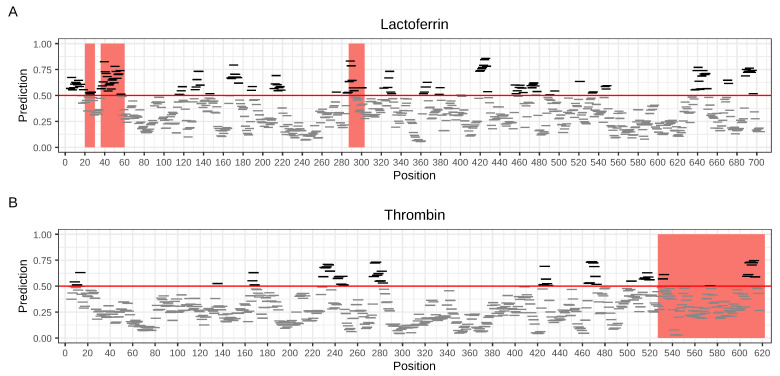
Distribution of 10-mers along the lactoferrin (**A**) and thrombin (**B**) sequences. AMP and non-AMP 10-mers were indicated by black and gray horizontal lines, respectively. The red line represents the cut-off value of 0.5. The red bars mark the fragments that have already been experimentally verified as AMPs: 1–11, 17–41 and 268–284 for lactoferrin [32] and 527–622, 597–622 and 604–622 for thrombin [37]; the sequence coordinates for lactoferrin do not include an N-terminal signal peptide (1–19).

As indicated in the benchmark section, AmpGram is the best AMP classifier that also robustly detects longer AMPs. Moreover, AmpGram predicts regions that have some antimicrobial potential. It scans a protein sequence with a sliding window of 10 amino acids in search of n-grams characteristic for AMPs and non-AMPs. Consequently, it divides the protein into overlapping subsequences of 10 amino acids (10-mers) that either are or are not AMPs (for details, see Section 3). The 10-mers are subsequently plotted along the sequence of the whole protein indicating regions that have strong antimicrobial potential. In Figure 3, exemplary results for lactoferrin (AmpGram prediction probability 0.627) and thrombin (AmpGram prediction probability 0.839) are presented.

In the case of lactoferrin, three regions have already been experimentally confirmed as AMPs, and two of them lactoferricin (17–41) and lactoferrampin (268–284) were clearly identified by AmpGram as AMPs [32]. Moreover, AmpGram detected many more regions in lactoferrin sequence that could represent potential AMPs. They can be easily identified in Figure 3A as sites with many overlapping AMP 10-mers (Table S6). Interestingly, the distribution of AMP 10-mers also perfectly reflects the evolutionary history of lactoferrin, i.e., its origin by a gene duplication event [48]. There are six distinct regions with the accumulation of AMP 10-mers: three in the N-terminal globular domain and three in the C-terminal one.

Human thrombin is a typical cryptic AMP. While it does not have any AMP properties, its C-terminal region does, and moreover the AMP fragments constitute a novel class of AMPs [37]. AmpGram prediction reveals that the AMP potential of the longest experimentally confirmed thrombin fragment (527–622) seems to be restricted to its C-terminus and overlaps with the other two shorter AMP fragments (597–622, 604–622). As in the case of lactoferrin, AmpGram also detected many more regions in thrombin that presumably could represent AMPs (Figure 3B; Table S6).

## 3. Materials and Methods

### 3.1. Datasets

In order to construct the positive, i.e., antimicrobial, dataset, 12,389 AMPs were retrieved from dbAMP [39], which is at present the most comprehensive database for AMPs. It includes information from other publicly available AMP databases, such as APD3 [44], CAMPR3 [27], ADAM [25], PhytAMP [49], AMPer [50], AntiBP2 [51], BACTIBASE [52] and LAMP [53]. Sequences containing nonstandard amino acids (B, J, O, U, X, Z) were removed from the positive dataset. In order to reduce the redundancy, and consequently bias in the antimicrobial dataset, sequence clustering was performed with CD-HIT program (version 4.8.1) at the identity threshold 0.90 [54]. In total, the final positive dataset contained 2463 peptides.

**Figure 4:**
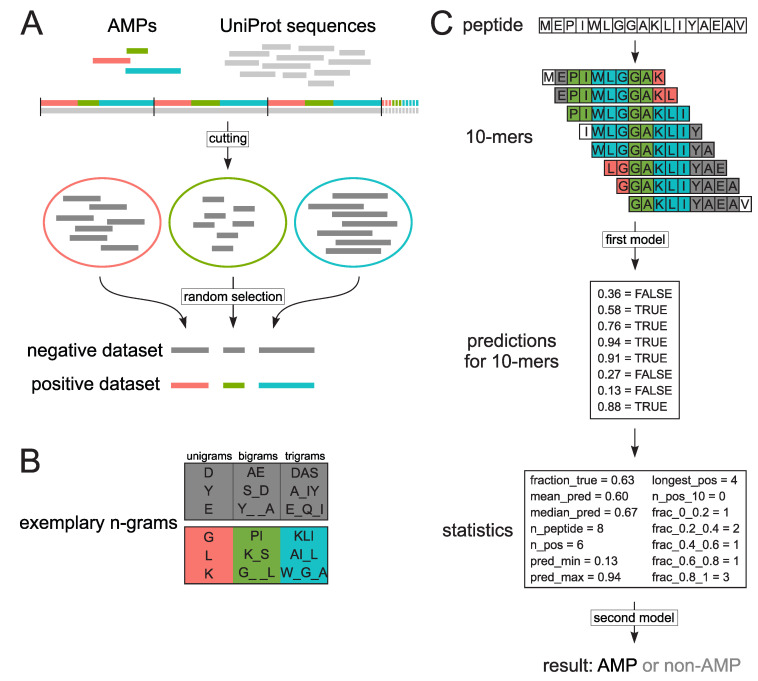
Schematic representation of datasets preparation (**A**), n-grams (**B**) and decision-making procedure in AmpGram (**C**). The positive dataset was constructed from sequences downloaded from the dbAMP database [39] (red, green and blue horizontal lines). To create the negative dataset, non-antimicrobial sequences (grey horizontal lines) were retrieved from the UniProt database [38]. The sequences were first concatenated into one string (grey horizontal line), and then cut (black vertical lines) into blocks corresponding in length to sequences from the positive dataset (red, green and blue horizontal line). The extracted blocks were next cut (not indicated in the figure) into subsets corresponding in length to sequences from the positive dataset (red, green and blue circles) and from them individual sequences were randomly selected for the negative dataset (**A**). Exemplary n-grams used to train AmpGram: the positive n-grams are shaded in red, green and blue, and the negative ones in grey (**B**). To make a prediction, AmpGram first divides a peptide into subsequences of 10 amino acids (10-mers). For each 10-mer, AmpGram makes a prediction if it is an AMP (true) or not (false) (first model). To scale the prediction for 10-mers to the whole peptide, a lot of statistics is calculated and on their basis AmpGram makes the final prediction (second model). Abbreviations of the statistics: Fraction_true--fraction of positive 10-mers, pred_mean–mean value of prediction, pred_median–median of prediction, n_peptide - number of 10-mers in a peptide, n_pos–number of positive 10-mers, pred_min–minimum value of prediction, pred_max–maximum value of prediction, longest_pos–the longest stretch of consecutively occurring 10-mers predicted as positive, n_pos_10–number of streches comprising of at least 10 10-mers predicted as positive, frac_0_0.2--fraction of 10-mers with prediction in range [0, 0.2], frac_0.2_0.4–fraction of 10-mers with prediction in range (0.2, 0.4], frac_0.4_0.6–fraction of 10-mers with prediction in range (0.4, 0.6], frac_0.6_0.8–fraction of 10-mers with prediction in range (0.6, 0.8], frac_0.8_1–fraction of 10-mers with prediction in range (0.8, 1]) (**C**).

As there are only few sequences verified as non-AMPs, the negative dataset was created using peptides extracted from cytoplasmic proteins similarly to datasets presented by Gabere and Noble [29]. We downloaded 544,249 sequences from UniProt (version from 20.12.2019) [38] that were experimentally validated as proteins without documented antimicrobial, antibacterial, antiviral or antifungal activity, and did not posses a mitochondrial or plastid transit peptide. We excluded proteins carrying mitochondrial or plastid transit peptides because their presequences were hypothesised to have evolved from AMPs [55], and therefore might have introduced bias in the negative dataset. The sequences downloaded from UniProt [38] were concatenated into a single string. From the concatenated string, we cut off blocks equal in length to all 2463 sequences from the positive dataset. Next, within the extracted blocks, we cut off sequences corresponding in length to AMPs from the randomly mixed positive dataset. For each AMP in the positive dataset, a subset of non-AMP sequences equal in size to a given AMP was created. Finally, from each subset of non-AMPs, we randomly collected one sequence for the negative dataset amounting to 2463 sequences (Figure 4A).

We divided both positive and negative dataset into five equally sized groups of sequence lengths: (i) 11–19, (ii) 20–26, (iii) 27–36, (iv) 37–60 and (v) 61–710, in order to ensure similar length distribution of sequences in the training and benchmark dataset. Next, we randomly extracted one tenth of sequences from each group to create the benchmark dataset. It comprised 247 AMP and 247 non-AMP sequences and was subsequently used to compare the performance of AmpGram with other top-ranking predictors. The remaining 2216 sequences in each dataset were used to train AmpGram.

We also compared the performance of AmpGram and other AMP predictors, including AMPScanner [24], on the benchmark datasets from Gabere and Noble [29]. They used 1713 AMP and 8565 non-AMP sequences from the APD3 database [44], and 547 AMP and 2735 non-AMP sequences from the DAMPD database [45]. To ensure the unbiased character of the DAMPD dataset in favour of AmpGram and AMPScanner [24], 336 AMP sequences were removed from the DAMPD dataset that were present either in the AmpGram (240 sequences) or AMPScanner (239 sequences) [24] training dataset. The benchmark without their removal is presented in the Supplementary Materials Figure S3 and Table S5.

### 3.2. Extraction of Encoded N-Grams

We scanned each sequence with a sliding window of 10 amino acids dividing it into overlapping subsequences of 10 amino acids (10-mers). All 10-mers from the positive dataset were considered as AMPs, whereas all 10-mers from the negative dataset as non-AMPs. Consequently, we obtained 87,716 AMP 10-mers and 87,599 non-AMP 10-mers. For each 10-mer in the positive and negative dataset, we extracted n-grams, which are continuous or discontinuous sequences of n elements. We considered unigrams (n-gram of size 1), bigrams (n-gram of size 2) and trigrams (n-gram of size 3), we separately analyzed continuous and discontinuous n-grams. For bigrams, we considered n-grams with a gap length from 1 to 3, whereas trigrams could contain only a single gap between the first and the second or the second and the third position. Next, the counts of n-grams were binarized, where 1 means that an n-gram was present in the sequence and 0 if it was absent (Figure 4B).

### 3.3. Model Training with Random Forests

The classifier with the best ability to correctly predict 10-mers with AMP activity was chosen during five-fold cross-validation using different length groups of sequences for training. The use of 11-26-amino-acid-long peptides, both 893 AMP and non-AMP sequences that resulted in 8791 AMP and 8818 non-AMP 10-mers, yielded the best results. We used random forests as the classification algorithm and trained them on the binarized n-grams extracted from 10-mers of the positive and negative dataset (Figure 4B,C). We considered only the most informative n-grams (13,087) selected by Quick Permutation Test (QuiPT) [40]. We grew the forest with 2000 trees and the default number of variables to possibly split at each node (rounded down square root of the total number of variables). To speed up the computation, we used the fastest implementation of random forests in R, i.e., the ranger package [56].

In order to scale the prediction for 10-mers to the whole peptide, we calculated the following statistics for each peptide using prediction for its 10-mers: (i) fraction_true–fraction of positive 10-mers, (ii) pred_mean–mean value of prediction, (iii) pred_median–median of prediction, (iv) n_peptide–number of 10-mers in a peptide, (v) n_pos–number of positive 10-mers, (vi) pred_min–minimum value of prediction, (vii) pred_max–maximum value of prediction, (viii) longest_pos–the longest stretch of consecutively occurring 10-mers predicted as positive, (ix) n_pos_10–number of stretches comprising of at least 10 10-mers predicted as positive, (x) frac_0_0.2–fraction of 10-mers with prediction in range [0,0.2], (xi) frac_0.2_0.4–fraction of 10-mers with prediction in range (0.2, 0.4], (xii) frac_0.4_0.6–fraction of 10-mers with prediction in range (0.4, 0.6], (xiii) frac_0.6_0.8–fraction of 10-mers with prediction in range (0.6, 0.8], (xiv) frac_0.8_1–fraction of 10-mers with prediction in range (0.8, 1]). The above statistics were used to train the second random forest model with the default value of number of trees (500) and mtry parameter (Figure 4C). The second random forest layer is responsible for deciding whether a given peptide (a collection of overlapping 10-mers) is an AMP or not. The following architecture is also known as the stacked random forest [57].

## 4. Conclusions

AmpGram is a novel AMP predictor that uses n-grams to represent information hidden in amino acid sequences and random forests as the classification algorithm. In comparison to other top-ranking AMP predictors, including AMPScanner, CAMPR3R and iAMPpred, AmpGram performs better at detecting AMPs. To the best of our knowledge, AmpGram is the first AMP classifier created for the prediction of longer AMPs and high-throughput proteomic screening. The application of n-grams made it possible to overcome the problem of high score–length dependency that was first indicated by Gabere and Noble [29] and also confirmed in our research. AmpGram not only allows to predict AMPs with high accuracy, but also precisely indicates peptide/protein fragments and regions that do have AMP potential. In order to test how AmpGram predictions relate to actual biological activity, we performed analyses for lactoferrin and thrombin; the former is a well-knownantimicrobial protein and the latter represents a cryptic AMP. Cryptic AMPs do not exhibit any AMP properties as mature proteins but their proteolytic products do. As expected, AmpGram identified both lactoferrin and thrombin as AMPs and indicated their potential AMP fragments and regions, including the sequences previously verified experimentally as AMPs [32,37]. The examples of lactoferrin and thrombin prove that antimicrobial fragments and regions predicted by AmpGram are good candidates for further investigation in terms of bactericidal activity, stability, toxicology, pharmacokinetics and the rational design of new AMPs; their antimicrobial activity can be further improved by amino acid modification to balance the peptide hydrophobicity and positive charge vital for disrupting bacterial membranes [58]. Moreover, the small size of AmpGram predicted fragments makes them easy to synthesize and exhibit potentially fewer side effects compared to longer AMPs [43].

AmpGram is available as a web server for multiple query sequences; however, for high-throughput proteomic screening, the users are encouraged to use its stand-alone version (see Appendix A). Therefore, we have also implemented AmpGram as an easy-to-use R package.

## Figures and Tables

**Table 1:** Peptide and protein length distribution in the UniProt [38] and dbAMP [39] database divided into length groups according to the AmpGram benchmark dataset (for details, see Section 3).

Length Range	UniProt	dbAMP
0.85 < 10	1119	508
11–19	1862	1894
0.8520–26	1016	1634
27–36	2439	1779
0.8537–60	9810	2049
61–710	482,852	4520
0.85 > 710	45,178	5

**Table 2:** Comparison of AmpGram performance with other top-ranking predictors. Programs that do not provide prediction probability are marked with asterisks.

Software	AUC	Precision	Sensitivity	Specificity
AmpGram	0.9062	0.8147	0.8543	0.8057
ADAM *	0.7186	0.6800	0.8259	0.6113
0.85AMPScanner V2	0.9641	0.9027	0.9393	0.8988
CAMPR3-ANN *	0.7854	0.7765	0.8016	0.7692
0.85CAMPR3-DA	0.8069	0.7286	0.8259	0.6923
CAMPR3-RF	0.8958	0.7782	0.9231	0.7368
0.85CAMPR3-SVM	0.8363	0.7664	0.8502	0.7409
iAMP-2L *	0.7895	0.8095	0.7571	0.8219
0.85iAMPpred (antibacterial)	0.9008	0.8115	0.8543	0.8016
iAMPpred (antifungal)	0.9009	0.8458	0.8219	0.8502
0.85iAMPpred (antiviral)	0.8397	0.7828	0.7733	0.7854

**Table 3:** Comparison of AmpGram performance with other top-ranking predictors for 61–710-amino-acid-long AMPs. Programs that do not provide prediction probability are marked with asterisks.

Software	AUC	Precision	Sensitivity	Specificity
AmpGram	0.8390	0.7736	0.8542	0.7500
ADAM *	0.6875	0.7812	0.5208	0.8542
AMPScanner V2	0.9049	0.7963	0.8958	0.7708
CAMPR3-ANN *	0.7083	0.7000	0.7292	0.6875
CAMPR3-DA	0.5221	0.5263	0.8333	0.2500
CAMPR3-RF	0.6048	0.5714	0.9167	0.3125
CAMPR3-SVM	0.6228	0.5733	0.8958	0.3333
iAMP-2L *	0.7292	0.9583	0.4792	0.9792
iAMPpred (antibacterial)	0.8229	0.7188	0.9583	0.6250
iAMPpred (antifungal)	0.8110	0.7333	0.9167	0.6667
iAMPpred (antiviral)	0.7476	0.6324	0.8958	0.4792

**Table 4:** Comparison of AmpGram and AMPscanner [24] performance on the APD dataset with other predictors from Gabere and Noble’s benchmark and according to their methodology [29]. The very low values of precision are due to the very large negative dataset used (for details, see Section 3).

Software	AUC	Precision	Sensitivity	Specificity
AmpGram	0.9723	0.5531	0.9515	0.8462
ADAM	0.8774	0.3236	0.9095	0.6198
AMPA	0.6394	0.4377	0.3917	0.8994
AMPScanner V2	0.9848	0.6657	0.9743	0.9022
CAMPR3-RF	0.9528	0.5337	0.9480	0.8343
CAMPR3-SVM	0.9202	0.4958	0.9060	0.8158
DBAASP	0.7723	0.6008	0.6281	0.9165
MLAMP	0.8397	0.4052	0.7560	0.7781

**Table 5:** Comparison of AmpGram and AMPscanner [24] performance on the DAMPD dataset with other predictors from Gabere and Noble’s benchmark and according to their methodology [29]. Sequences used to train either AmpGram or AMPScanner were removed from the dataset. The benchmark without their removal is presented in Table S5 in the Supplementary Materials. The very low values of precision are due to the very large negative dataset used (for details, see Section 3).

Software	AUC	Precision	Sensitivity	Specificity
AmpGram	0.9321	0.3045	0.8673	0.8472
ADAM	0.7494	0.1540	0.7299	0.6907
AMPA	0.6813	0.2136	0.5355	0.8479
AMPScanner V2	0.9088	0.3661	0.8483	0.8867
CAMPR3-RF	0.8162	0.1991	0.8815	0.7265
CAMPR3-SVM	0.7862	0.1926	0.8626	0.7210
DBAASP	0.5165	0.1014	0.1043	0.9287
MLAMP	0.6833	0.1695	0.4692	0.8227

## Supplementary Materials

The following are available online at https://www.mdpi.com/1422-0067/21/12/4310/s1, **Figure S1** Amino acid composition of AMP and non-AMP sequences. The analysis was performed on sequences from positive and negative dataset, respectively (for details, see Section 3). The shorter the sequence, the stronger the differences in amino acid composition between AMPs and non-AMPs. For longer AMPs, i.e., over 60 amino acids, the differences between the datasets are hardly visible. **Figure S2** Comparison of AmpGram performance with other top-ranking predictors for (i) all AMP lengths and (ii) 11–19, (iii) 20–26, (iv) 27–36, (v) 37–60 and (vi) 61–710-amino-acid-long AMPs. **Figure S3** Comparison of AmpGram and AMPscanner [24] performance on the APD and DAMPD dataset with other predictors from Gabere and Noble’s benchmark and according to their methodology [29]. **Table S1** Comparison of AmpGram performance with other top-ranking predictors for 11–19-amino-acid-long AMPs. Programs that do not provide prediction probability are marked with asterisks. **Table S2** Comparison of AmpGram performance with other top-ranking predictors for 20–26-amino-acid-long AMPs. Programs that do not provide prediction probability are marked with asterisks. **Table S3** Comparison of AmpGram performance with other top-ranking predictors for 27–36-amino-acid-long AMPs. Programs that do not provide prediction probability are marked with asterisks. **Table S4** Comparison of AmpGram performance with other top-ranking predictors for 37–60-amino-acid-long AMPs. Programs that do not provide prediction probability are marked with asterisks. **Table S5** Comparison of AmpGram and AMPscanner [24] performance on the APD and DAMPD datasets with other predictors from Gabere and Noble’s benchmark and according to their methodology [29]. **Table S6** List of antimicrobial 10-mers for lactoferrin and thrombin, including experimentally confirmed fragments, predicted by AmpGram.

## Abbreviations

The following abbreviations are used in this manuscript:

AMP	Anti-microbial peptide

## Appendix A. Availability and Implementation

The code necessary to reproduce the analysis presented in this paper is available in the repository: https://github.com/michbur/AmpGram-analysis.

The AmpGram prediction web-server is available at: biongram.biotech.uni.wroc.pl/AmpGram.

AmpGram is implemented as an R package available at: https://CRAN.R-project.org/package=AmpGram.

The stand-alone version is dedicated for high-throughput proteomic screening.
